# Denervation of the paraspinal muscles in patients with scoliosis secondary to Chiari malformation and syringomyelia: does it improve following posterior fossa decompression?

**DOI:** 10.1186/1748-7161-10-S1-O11

**Published:** 2015-01-19

**Authors:** Zezhang Zhu, Shifu Sha, Xu Sun, Zhen Liu, Huang Yan, Weiguo Zhu, Jun Jiang, Yong Qiu

**Affiliations:** 1 Spine Surgery, Drum Tower Hospital of Nanjing University Medical School, Nanjing, China

**Keywords:** Chiari malformation, Syringomyelia, Scoliosis, Posterior fossa decompression, Paraspinal muscle, Denervation

## Objective

To determine whether denervation of the paraspinal muscles would improve following posterior fossa decompression (PFD) in patients with scoliosis secondary to Chiari malformation and syringomyelia through evaluating the alterations in expression of bax and bcl-2, two genes known to be pivotal for the regulation of cellular apoptosis.

## Methods

Fourteen patients with scoliosis secondary to Chiari malformation and syringomyelia treated between July 2011 and July 2012 were prospectively enrolled. Bilateral biopsy of paraspinal muscles was performed during PFD and subsequent scoliosis surgery. Bax and bcl-2 protein levels were examined by Western blotting and then quantitatively assessed using a scanning densitometer.

## Results

The initial age and primary curve magnitude averaged 16.0 ± 3.3 years and 63.8° ± 18.3°, respectively. At 7.6 ± 2.6 months post-PFD, significant decreases in mean net gray value, positive area and positive ratio were noted for the 20kd bax protein (P=0.021, 0.013 and <0.001, respectively). The bcl-2 protein, in contrast, appeared to be enriched over the same period in protein lysates. Specifically, the positive area increased from 10.6 ± 6.1 (10^4^) to 21.3 ± 9.2 (10^4^) (P=0.001), while the positive ratio increased from 0.40 ± 0.17 to 0.85 ± 0.19 (P<0.001). Regarding the net gray value, a similar upward trend was observed though not reaching statistical significance (84.4 ± 35.8 versus 101.6 ± 33.3, P =0.197).

## Conclusion

In patients undergoing PFD for Chiari malformation and syringomyelia, myocyte apoptosis could be inhibited through down-regulation of bax and up-regulation of bcl-2, indicating an improvement in denervation of the paraspinal muscles.

